# Characterization of the chloroplast genome of *Cassia siamea* Lain, a rosewood species from southeast China

**DOI:** 10.1080/23802359.2019.1692717

**Published:** 2019-11-20

**Authors:** Zhou Hong, Kun-Kun Zhao, Ning-Nan Zhang, Zeng-Jiang Yang, Xiao-Jin Liu, Da-Ping Xu

**Affiliations:** State Key Laboratory of Tree Genetics and Breeding, Research Institute of Tropical Forestry, Chinese Academy of Forestry, Guangzhou, China

**Keywords:** *Cassia siamea*, rosewood, chloroplast genome, phylogenetic analysis

## Abstract

*Cassia siamea* is a rosewood species in Southwest China with high wood and medicinal value. To clarify genetic background of *C. siamea*, we sequenced chloroplast genome by Illumina Hiseq and PacBio Sequel. The whole genome was 148,437 bp in length, containing a large single copy region (77,723 bp), a small single copy region (18,462 bp) and a pair of inverted repeats regions (26,126 bp). The cp genome contained 102 genes (71 protein-coding genes, 27 tRNAs and 4 rRNAs). The phylogenetic analysis indicated that *C. siamea* is close to *Senna tora* within Cassiinae/Caesalpiniaceae. The complete chloroplast genome of *C. siamea* will provide useful resources for the development and utilization of this species and the phylogenetic study of Fabaceae.

*Cassia siamea* is one of the tree species of the genus *Cassia* within the family Caesalpiniaceae. It is widely distributed in southwest of China such as Yunnan, Guangdong, Hainan, Guangxi, and Fujian provinces (China Flora Editorial Board, Chinese Academy of Sciences 2006). The heartwood and leaf of this species contains a series of chemical components, with the functions of antitumor, anti-plasmodium and antiviral for HIV (Kashiwada et al. 1996; Ajaiyeoba et al. 2008). With the advantage of its fastgrowth, strong germination ability and high wood quality, *C. siamea* was planted as a high-grade rosewood (Thapa and Subedi 2017). Due to its high medicinal and commercial value, *C. siamea* has been overexploited for a long time and has been listed in the Chinese National Stardard “Hongmu” (GB/T 18107-2017). In recent years, with the restriction of origin countries on the export of rosewood and the exhaustion of global rosewood resources, the price of *C. siamea* increased quickly. However, there is only molecular study of this species were carried on DNA extraction and species identification of wood tissue from *C. siamea* by DNA-ITS (Li et al. 2015).

The cpDNA was extracted from fresh leaves of *C. siamea* were collected from a 30-years old tree, which grew in rosewood national forest tree germplasm resources repository, Ledong District, Hainan Province, China (18°41′24″N, 108°47′24″E) (McPherson et al. 2013), and was stored in the Herbarium of Research Institute of Tropical Forestry, Chinese Academy of Forestry (accession number: CP-CS20190608). High-throughput sequencing was carried out on Illumina HiSeq and PacBio RS II in Shanghai Biozeron Biotech CO., Ltd (Shanghai, China) (Borgstrom et al. 2011). The chloroplast genes and genome map of the *C. siamea* were annotated by the DOGMA (Dual Organellar GenoMe Annotator) (Wyman et al. 2004) and OGDRAW (Organellar Genome DRAW) (Lohse et al. 2007), respectively. Whole cp genome of 18 Fabaceae including *C. siamea* were constructed phylogenetic analysis by the maximum-likelihood (ML) methods using PhyML 3.0 (Guindon et al. 2010).

The complete and annotated cp genome sequence of *C. siamea* (MN525772) was a circular molecule 148,437 bp, included a quadripartite structure that consists of a large single copy (LSC) region of 77,723bp and a small single copy (SSC) region of 18,462 bp, separated by a pair of inverted repeat (IR) regions of 26,126 bp. The overall GC content was 36.31%. While the corresponding values of the LSC, SSC, and IR regions were 34.81%, 31.82%, and 42.98%, separately. It encoded 102 genes, including 71 protein-coding genes, 27 tRNAs and 4 rRNAs. Among these, only three genes possessed two introns, the other nine genes contained a one intron. Five PCGs, 7 tRNAs genes, and 4 rRNAs genes were duplicated in both IR regions. There was one PCG (*ycf1*) in IRb and SSC region, and the other PCG (*rps19*) in LSC and IRb region.

Phylogenetic trees showed that *C. siamea* is close to *Senna tora* within Cassiinae/Caesalpiniaceae (Figure 1). In this study, we reconstructed the complete chloroplast genome of *C. siamea* and phylogenetic relationships within Leguminosae to provide the underlying information for conservation works on *C. siamea* as well as for evolutionary and phylogenetic studies within Caesalpiniaceae, even Leguminosae.

**Figure 1.**
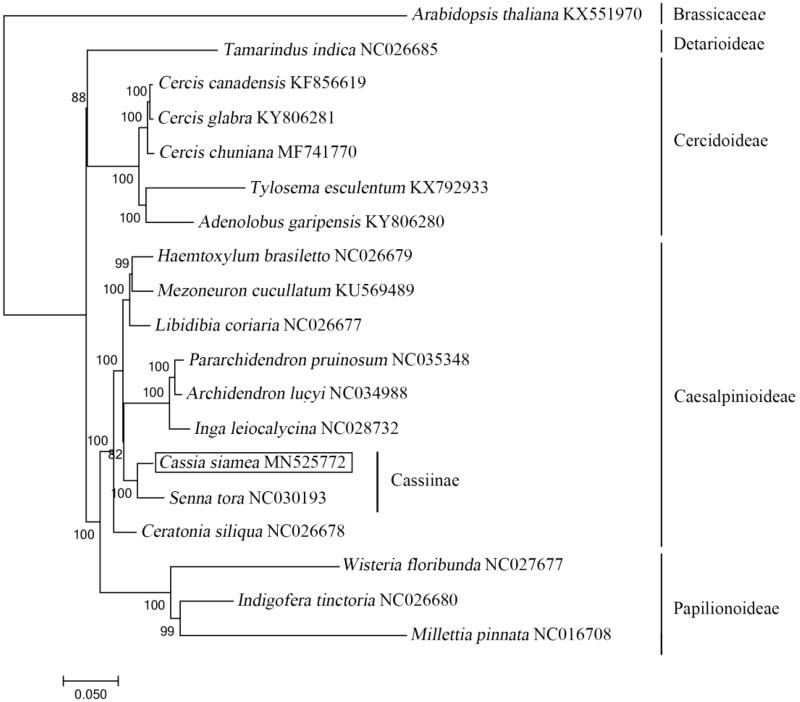
Maximum-likelihood phylogenetic tree inferred from 19 cp genomes. The position of *Cassia siamea* is shown in a box and bootstrapping values are listed for each node. *Arabidopsis thaliana* as outgroup species.
